# Concurrent Spontaneous Sublingual and Intramural Small Bowel Hematoma due to Warfarin Use

**DOI:** 10.1155/2015/583869

**Published:** 2015-11-16

**Authors:** Gül Pamukçu Günaydın, Hatice Duygu Çiftçi Sivri, Serkan Sivri, Yavuz Otal, Ayhan Özhasenekler, Gülhan Kurtoğlu Çelik

**Affiliations:** ^1^Department of Emergency Medicine, Ankara Atatürk Training and Research Hospital, Çankaya, 06800 Ankara, Turkey; ^2^Department of Cardiology, Ankara Atatürk Training and Research Hospital, Çankaya, 06800 Ankara, Turkey; ^3^Department of Emergency Medicine, Faculty of Medicine, Yıldırım Beyazıt University, Çankaya, Ankara, Turkey

## Abstract

*Introduction*. We present a case of concurrent spontaneous sublingual and intramural small bowel hematoma due to warfarin anticoagulation. *Case*. A 71-year-old man presented to the emergency department complaining of a swollen, painful tongue. He was on warfarin therapy. Physical examination revealed sublingual hematoma. His international normalized ratio was 11.9. The computed tomography scan of the neck demonstrated sublingual hematoma. He was admitted to emergency department observation unit, monitored closely; anticoagulation was reversed with fresh frozen plasma and vitamin K. 26 hours after his arrival to the emergency department, his abdominal pain and melena started. His abdomen tomography demonstrated intestinal submucosal hemorrhage in the ileum. He was admitted to surgical floor, monitored closely, and discharged on day 4. *Conclusion*. Since the patient did not have airway compromise holding anticoagulant, reversing anticoagulation, close monitoring and observation were enough for management of both sublingual and spontaneous intramural small bowel hematoma.

## 1. Introduction

Sublingual hematoma is a rare and potentially life threatening complication of oral anticoagulation [1–3]. Spontaneous intramural small bowel hematoma (SISBH) due to oral anticoagulation is also rare but generally is not life threatening and may improve with medical treatment [4].

We present the case of concurrent spontaneous sublingual and intramural small bowel hematoma due to warfarin anticoagulation. To our knowledge, there is only one published case in Google scholar and none in PubMed that these two entities are seen together.

## 2. Case

A 71-year-old man presented to the emergency department complaining of a swollen, painful tongue and difficulty to speak that began a few hours ago. He did not have recent trauma, dental work, cough, or fever. He was on warfarin therapy, 5 mg/day for recurrent deep vein thrombosis for over a year.

**Figure 1:**
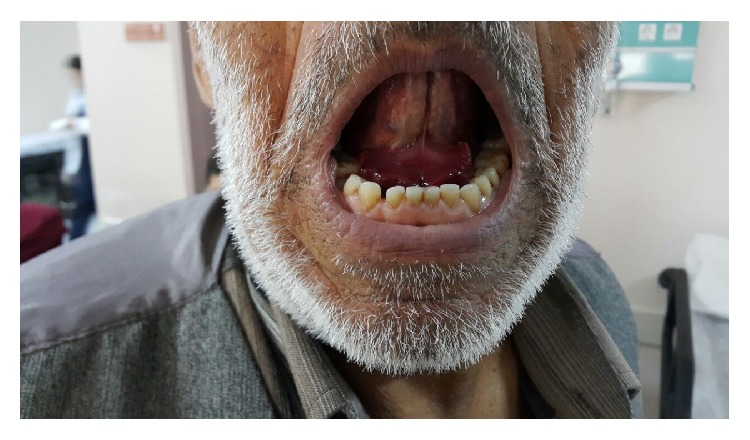
Sublingual hematoma.

**Figure 2:**
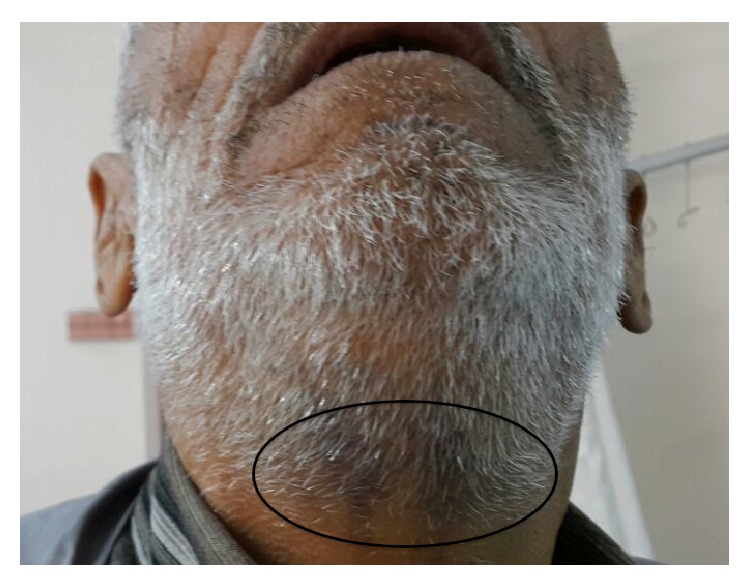
Submental ecchymosis.

His vital signs were within normal limits. Oral physical examination revealed 3 × 3 cm sized, red-purple, tense, and tender mass consistent with a sublingual hematoma (Figure 1). He was not in respiratory distress and did not have stridor. The submental triangle was swollen and ecchymotic (Figure 2).

Laboratory studies were significant for international normalized ratio (INR) of 11.9 (0.8–1.2), with a prothrombin time (PT) of 139.1 sec (8.8–14), and with partial thromboplastin time (PTT) of 108.7 sec (22–38). He said that his INR levels were not checked for over 6 months because he was living in a remote village that was 2 hours away from the nearest hospital that has the test. His hemoglobin was 14.2 g/dL, platelet count was 234 K/*μ*L, and white blood cell count was 15.7 K/*μ*L. His electrolytes, liver function tests, urea, and creatine levels were within normal limits. The computed tomography scan of the neck demonstrated sublingual hematoma.

The patient was evaluated by otolaryngologist. Flexible nasopharyngoscopy demonstrated that the hematoma was limited to the sublingual area. We stopped warfarin treatment and coagulopathy was reversed with 10 mg of IV vitamin K and 5 units of fresh frozen plasma. An INR measurement was 1.4 after 3 hours. He was given fentanyl for pain reduction.

**Figure 3:**
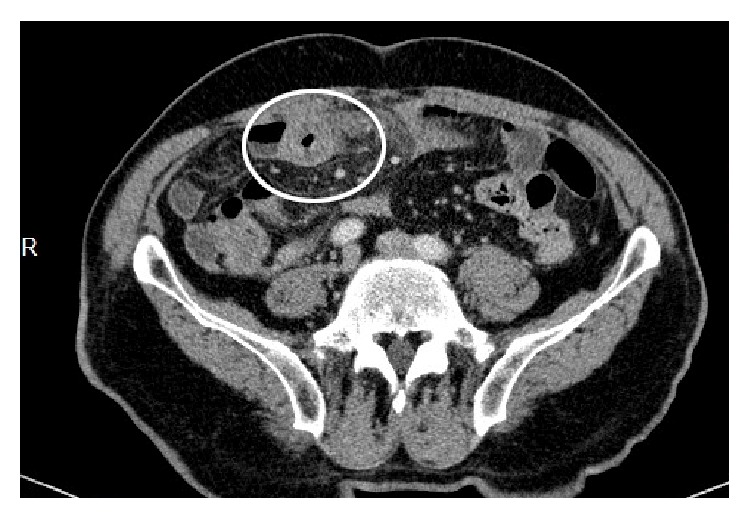
Intestinal submucosal hemorrhage.

26 hours after his arrival to the emergency department, his abdominal pain and melena started. Abdominal ultrasound revealed mural thickening in the intestinal wall. His abdomen tomography demonstrated intestinal submucosal hemorrhage in the ileum (Figure 3).

The patient was evaluated by general surgeon and was admitted. He was monitored closely and kept nothing by mouth. He was started on IV fluids and omeprazole. His hematocrit remained stable, and both sublingual and intestinal hemorrhage resolved slowly. He was started on oral diet on day 3 and discharged on day 4.

## 3. Discussion

Bleeding is the main complication of anticoagulation [5]. The incidence of hemorrhage is related to INR level [1]. Our patient had elevated INR level possibly due to not being regularly checked since he was living in village far away from the hospital.

Upper airway hematomas are rare. Retropharyngeal, submaxillary and epiglottic hematomas may be difficult to diagnose, but sublingual hematoma can be seen easily [2]. Computed tomography of the upper airway is needed to define the extent of hemorrhage [6, 7]. In our patient physical exam revealed sublingual hematoma. CT was obtained to see the extent of hemorrhage.

Sublingual hematoma may be life threatening by causing airway obstruction. Some authors recommend that medical treatment combined with early prophylactic surgical airway should be standard of care but there is no consensus [8]. Surgical decompression of the hematoma is a treatment choice but it may cause edema, airway obstruction, and massive bleeding [9]. Awake fiber optic nasotracheal intubation is a good option for securing the airway; it can also show the extension of hemorrhage into other areas of upper airway [8]. Since the patient did not have airway compromise he was observed conservatively and we did not perform surgery or early intubation or surgical airway.

Medical treatment of overanticoagulation is withholding the drug, parenteral vitamin K and fresh frozen plasma or factor concentrates [1, 2, 10]. Some researchers recommend the use of antibiotics in the treatment of upper airway hematomas because it may be a result of localized infection and vasodilatation [1] but our patient did not have any symptoms of infection and antibiotics were not necessary. The use of systemic steroids is controversial and not of proven benefit in upper airway hematomas [1]; we did not use steroids.

The clinical presentation of SISBH depends on the location and extent of the hematoma. Acute abdominal pain and ileus symptoms in patient on anticoagulation treatment should raise suspicion. USG may show intramural bowel hematomas but its sensitivity is less than CT [11]. CT may show circumferential bowel wall thickening, intramural hyper density, luminal narrowing, and intestinal obstruction. In our patient the diagnosis was made with USG and CT was obtained to see the exact location and extent of hematoma.

Holding anticoagulant, reversing anticoagulation, close monitoring and observation will be enough in management of most SISBH cases [11]. Surgery should be reserved for cases with uncertain diagnosis or complications like intra-abdominal hemorrhage, perforation, peritonitis, and intestinal obstruction not responding to conservative treatment [12]. Our patient was monitored closely and reversal of anticoagulation was enough for successful treatment. Since melena was self-limited and it was not accompanied by hematemesis we think it may be due to the penetration of the intestinal hematoma that eventually healed by supportive treatment.
